# Safety and tolerability of topically administered autologous, apoptotic PBMC secretome (APOSEC) in dermal wounds: a randomized Phase 1 trial (MARSYAS I﻿)

**DOI:** 10.1038/s41598-017-06223-x

**Published:** 2017-07-24

**Authors:** Elisabeth Simader, Denise Traxler, Mohammad Mahdi Kasiri, Helmut Hofbauer, Michael Wolzt, Christoph Glogner, Angela Storka, Michael Mildner, Ghazaleh Gouya, Alexandra Geusau, Carola Fuchs, Claudia Eder, Alexandra Graf, Michaela Schaden, Bahar Golabi, Marie-Bernadette Aretin, Susanne Suessner, Christian Gabriel, Walter Klepetko, Erwin Tschachler, Hendrik Jan Ankersmit

**Affiliations:** 1 Division of Thoracic Surgery, Medical University of Vienna, Vienna, Austria; 2 Christian Doppler Laboratory for Cardiac and Thoracic Diagnosis and Regeneration, Medical University of Vienna, Vienna, Austria; 3 Department of Clinical Pharmacology, Medical University of Vienna, Vienna, Austria; 4 Research Division of Biology and Pathobiology of the Skin, Department of Dermatology, Medical University of Vienna, Vienna, Austria; 5 Division of Immunology, Allergy, and Infectious Diseases, Department of Dermatology, Medical University of Vienna, Vienna, Austria; 6 Section of Medical Statistics, Centre for Medical Statistics, Informatics and Intelligent Systems, Medical University of Vienna, Vienna, Austria; 7 Clinical Trials Coordinative Centre, Medical University of Vienna, Vienna, Austria; 8 Austrian Red Cross Blood Transfusion Service for Upper Austria, Linz, Austria; 9 FFG Project 852748 “APOSEC“, Medical University of Vienna, Vienna, Austria; 10 Aposcience AG, Company No.: 308089, Vienna, Austria; 11 AKH Vienna pharmacy, Medical University of Vienna, Vienna, Austria; 12 Danubian Hospital, Social and medical center of eastern Vienna, Vienna, Austria; 13 Ludwig Boltzmann Institute for experimental and clinical traumatology, Vienna, Austria

## Abstract

Developing effective therapies against chronic wound healing deficiencies is a global priority. Thus we evaluated the safety of two different doses of topically administered autologous APOSEC, the secretome of apoptotic peripheral blood mononuclear cells (PBMCs), in healthy male volunteers with artificial dermal wounds. Ten healthy men were enrolled in a single-center, randomized, double-blinded, placebo-controlled phase 1 trial. Two artificial wounds at the upper arm were generated using a 4-mm punch biopsy. Each participant was treated with both topically applied APOSEC and placebo in NuGel for 7 consecutive days. The volunteers were randomized into two groups: a low-dose group (A) receiving the supernatant of 12.5 × 10^6^ PBMCs and a high-dose group (B) receiving an equivalent of 25 × 10^6^ PBMCs resuspended in NuGel Hydrogel. Irradiated medium served as placebo. The primary outcome was the tolerability of the topical application of APOSEC. All adverse events were recorded until 17 days after the biopsy. Local tolerability assessment was measured on a 4-point scale. Secondary outcomes were wound closure and epithelization at day 7. No therapy-related serious adverse events occurred in any of the participants, and both low- and high-dose treatments were well tolerated. Wound closure was not affected by APOSEC therapy.

## Introduction

The global incidence of non-healing wounds is soaring due to increasing prevalence of diabetes and obesity. These wounds are a major cause of morbidity, have a negative impact on quality of life, and result in enormous costs for the health care system^1^. Although several highly expensive products are on the market, the process of wound healing takes a long time and is often incomplete, entailing amputation in severe cases^2^.

Many approaches to new therapies have been investigated over the last decades, but no sufficient therapeutic option yet exists. Wound healing involves a complex interplay of various cell types as well as cellular and biochemical events. This process depends on a supply of oxygen, nutrients, and growth factors. Diabetic patients have an impaired vasculature, which results in reduced blood perfusion to the wound area, leading to decreased migration of inflammatory cells^3, 4^. However, inflammatory cells are an essential part of chronic wound healing, acting in both beneficial and harmful ways^5–7^. The application of stem cells, genetically modified cells, or paracrine factors on chronic wound areas has led to encouraging results, regarding wound healing^8–10^. A paper by Holzinger *et al*. showed that topical application of activated, autologous peripheral blood mononuclear cells (PBMCs) effectively initiated epithelialization of ulcerated, dermal wounds and that wound closure was present in 92% of patients after 60 days, compared to standard therapy^11^. In particular, paracrine factors are being considered as a promising option because they provide pro-angiogenic and anti-apoptotic mediators for cell proliferation and migration^12^. To advance Holzinger’s “activated PBMC-based therapy,” we applied the cell-free secretome of apoptotic PBMCs, the apoptotic PBMC secretome (APOSEC), produced according to good manufacturing practice (GMP) guidelines. APOSEC contains a myriad of cytokines, lipids, proteins, exosomes, and vasoactive substances^13^. To increase the secretory output of PBMCs, we induced apoptosis via ionizing radiation^13–17^.

In a recent publication, we reported positive effects of APOSEC on angiogenesis and skin regeneration in a mouse wound-healing model and in a clinically more relevant porcine third-degree burn model^10, 14^. Further approaches in preclinical models revealed that the secretome of PBMCs attenuates hypoxic injury in acute and latent myocardial infarction^15, 18–20^, spinal cord injury, and stroke^21, 22^. Additionally, APOSEC augments de novo secretion of antimicrobial peptides^23^ and attenuates experimental myocarditis by inducing caspase 8–dependent CD4 T cell apoptosis^19^. These promising preclinical data encouraged us to initiate the production of APOSEC for human application under the auspices of the Austrian Agency for Food and Drug Safety (AGES) (AGES-Nr. INS-480102-0013-007). APOSEC as a drug substance has been classified as “biological” and can be applied in a personalized manner (autologous) or in an allogeneic approach (pooled product). This first clinical trial using autologous APOSEC was approved by the certified authority (AGES) to explore its safety and tolerability in artificial skin wounds in healthy, male participants.

## Materials and Methods

### Trial design and study population

**Figure 1:**
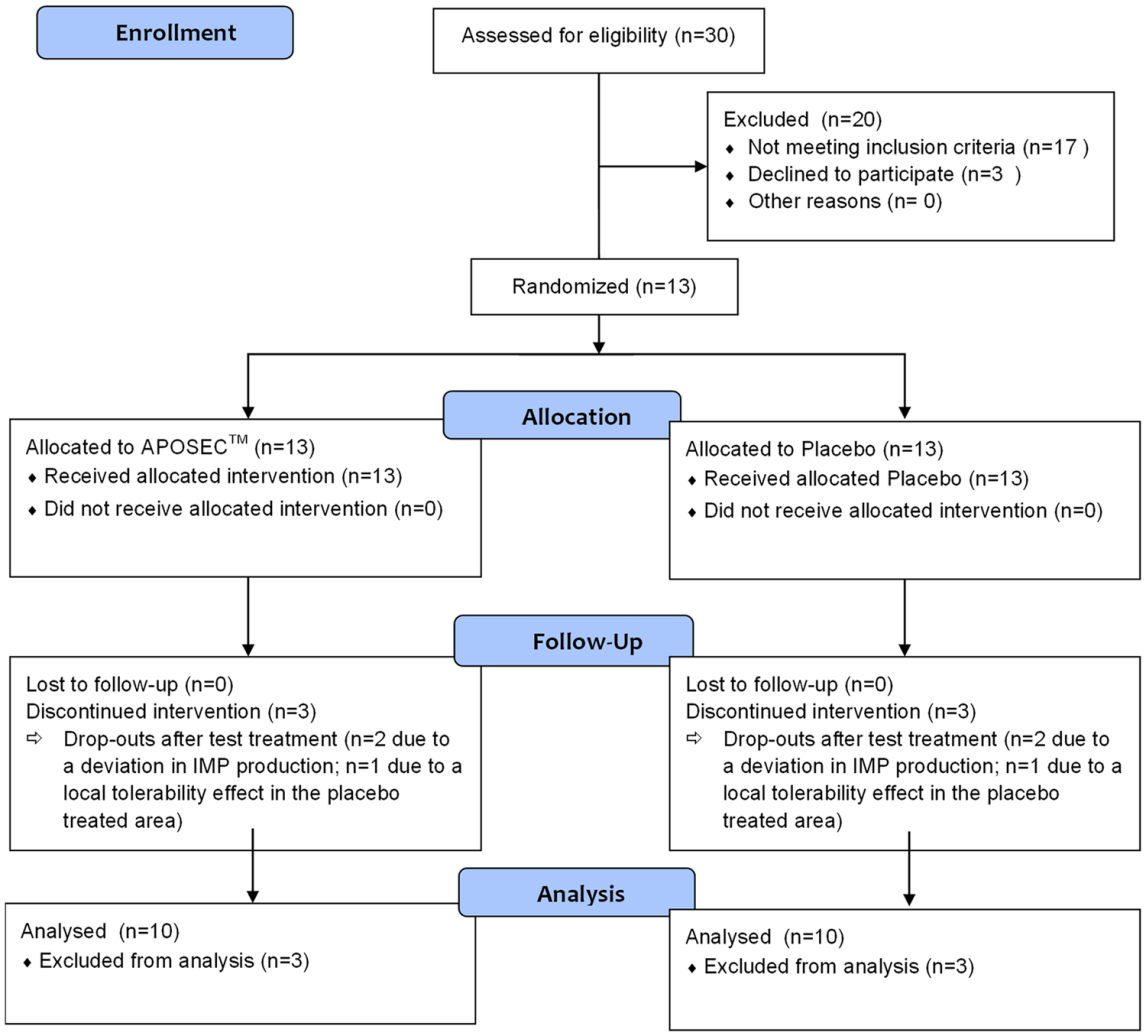
CONSORT Study Design of MARSYAS. The screening and design of the study were developed and conducted by the Department of Clinical Pharmacology of the Medical University of Vienna. Ten participants were included after giving written informed consent. Allocation to the low-dose group A and high-dose group B was completed after an interim analysis. To avoid inter-individual differences, every study participant received both verum and placebo on different positions on the same arm. The randomization of verum and placebo to the proximal or distal artificial arm wound was performed in a 1:1 ratio.

This study was a prospective, single-center, randomized, double-blinded, placebo-controlled, dose-finding phase 1 trial to assess the safety and tolerability of two different doses of autologous APOSEC in artificial dermal wounds. A secondary potential objective was to investigate the effect on wound closure. The study population consisted of 10 healthy male volunteers. Five participants were assigned to each group: a low-dose group (GMP APOSEC from 12.5 × 10^6^ irradiated, lyophilized PBMC/ml) and a high-dose group (GMP APOSEC from 25 × 10^6^ irradiated lyophilized PBMC/ml). (Supplementary Table S1 in the Supplement). Medium served as placebo. Both APOSEC and placebo were applied on two artificial dermal wounds (proximal and distal) on the upper non-dominant arm of the participant to reduce intra-individual reactions to a minimum (Fig. 1). (Exclusion criteria can be accessed at ClinicalTrials.gov Identifier: NCT02284360; https://clinicaltrials.gov/ct2/show/NCT02284360).

### Trial registration

EudraCT-Number: 2013-000756-17, NCT 02284360, AGES INS-480102-0013-007 https://clinicaltrials.gov/ct2/show/NCT02284360?term=02284360&rank=1, ClinicalTrials.gov Identifier:NCT02284360 (First received: October 30, 2014; Last updated: September 25, 2015; Last verified: September 2015).

#### Screening/run-in phase

**Figure 2:**
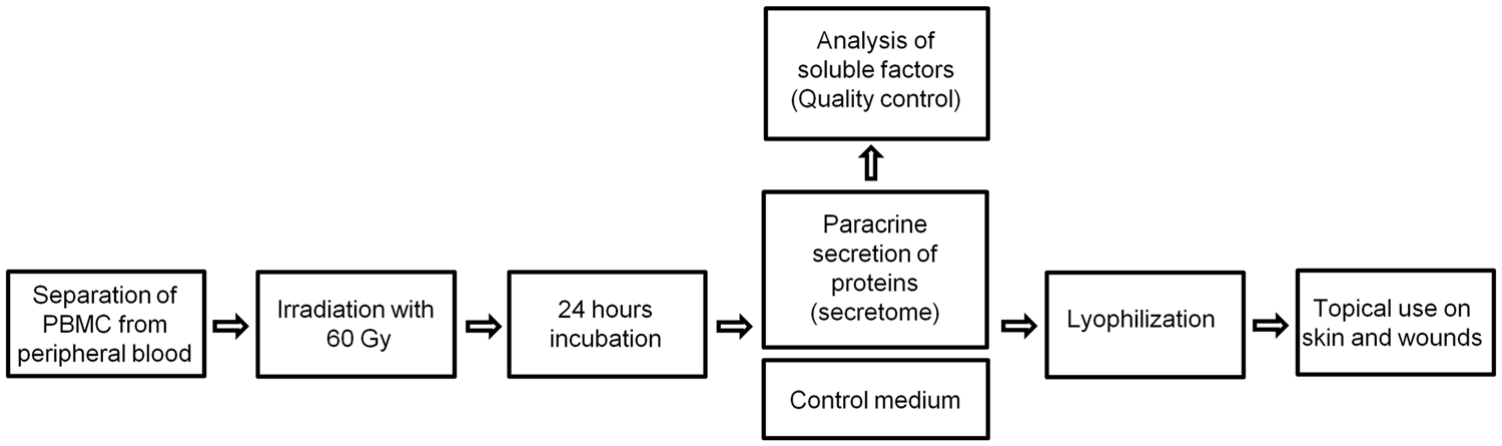
APOSEC production. Preparation process of APOSEC according to good manufacturing practice (GMP) in the facility of the Austrian Red Cross Blood Transfusion Service of Upper Austria (Linz, Austria), with the following steps. The first step was separating the PBMCs from the whole blood samples, inducing apoptosis via ionizing irradiation, and incubating for 24 h. During this 24 h, the PBMCs secrete a multitude of cytokines and chemokines. The quantity of cytokines is measured using ELISA and immunoassay (Luminex®100IS) for quality control. After the lyophilization, APOSEC is ready for topical use on skin and wounds.

After eligible study volunteers gave written informed consent, clinical and laboratory testing was performed to verify inclusion and exclusion criteria. Physical examination and vital signs were obtained and a standard 12-lead ECG was performed. Blood samples for hematology, serum chemistry, virology and urine samples for urine analysis were obtained. Demographic and medical history data as well as concomitant medication were assessed. Before initiation of the treatment phase, 450 ml blood was collected at the GMP facility at the Austrian Red Cross Blood Transfusion Service of Upper Austria (Linz, Austria) (AGES INS-480102-0013-007), and autologous APOSEC, was produced according to GMP guidelines (Fig. 2). Afterwards, APOSEC was transferred to the Pharmacy of the Medical University of Vienna by Med Log courier.

#### Randomization/treatment phase

Randomization and blinding were performed by the AKH Vienna pharmacy (Vienna, Austria). To reduce potential adverse events resulting from the investigative medicinal product (IMP) or wound dressing (Tegaderm Film 10 × 12 cm, 3 M, Maplewood, MN, US), a blinded test treatment with APOSEC and placebo on intact skin of the inner upper dominant arm was performed 24 h before initiation of the treatment phase. Any study participants who developed adverse events were excluded from the treatment phase. If adverse events were not considered to be IMP related, these volunteers were replaced. (Supplementary Table S2 in the Supplement).

Lyophilized APOSEC or the culture medium CellGro was resuspended in 200 μl 0.9% NaCl until complete dissolution, followed by mixing with 800 μl NuGel Hydrogel (Systagenix, Gatwick, West Sussex, UK) for topical administration only. The so produced verum or placebo was supplied in single-use tubes as a sterile preserved white gel.

**Figure 3:**
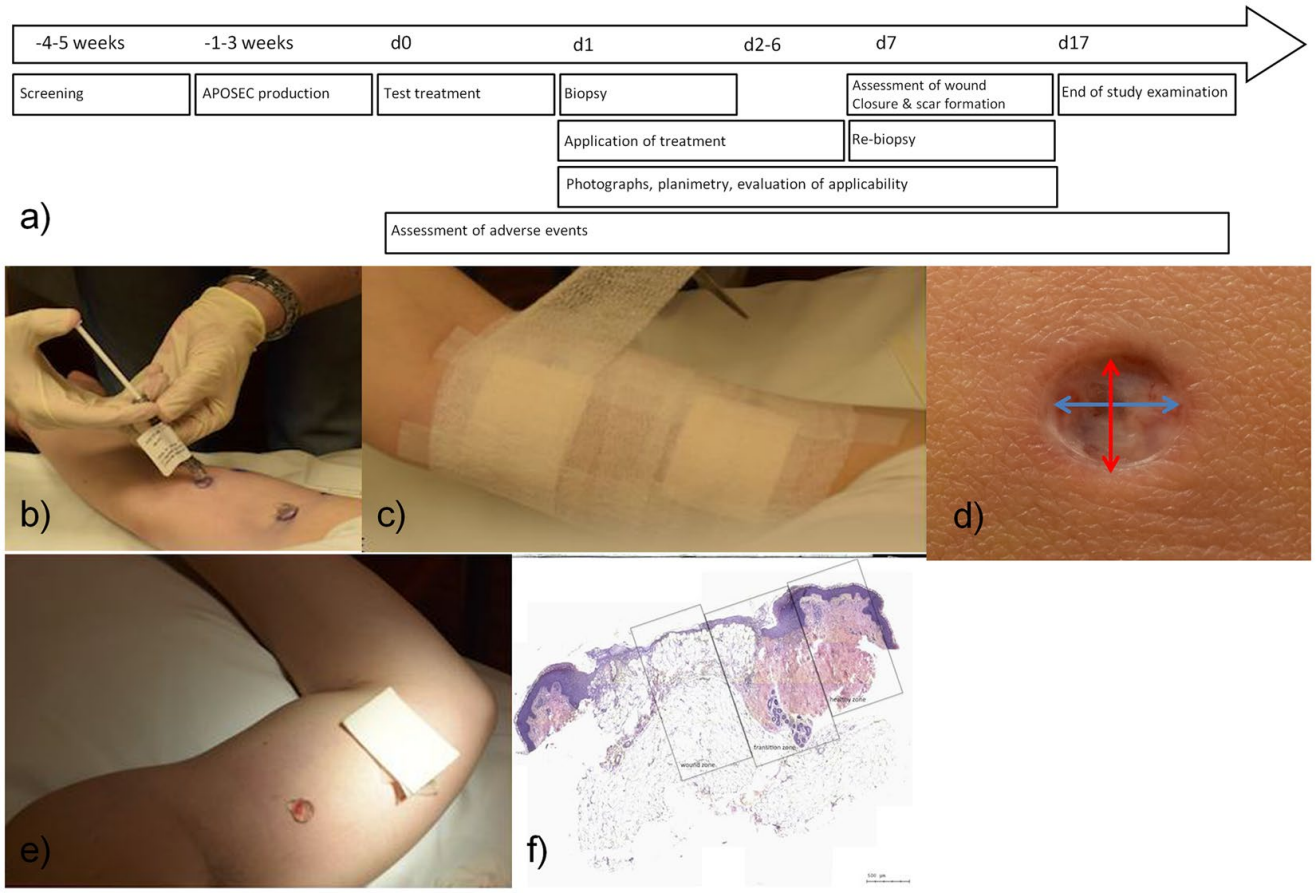
Study timeline and application of APOSEC/placebo. Study timeline (**a**). Application of APOSEC/placebo on intact skin (test treatment) (**b**). Bandaging of study site (**c**). Maximal (blue line) and minimal (red line) diameter of biopsy wound (**d**). Applied IMP/placebo on artificial wound (**e**). Tissue sample from day 7, boxes show wound, transition, and healthy zones in which measurements were performed (**f**).

Artificial wounds were generated by two 4-mm punch biopsies (distal and proximal, respectively) on the inner upper side of the non-dominant arm under local anesthesia. The distance between both biopsies was at least 8 cm. After being cleaned with 0.9% NaCl, one wound was treated with approximately 1 ml of APOSEC and the second with approximately 1 ml of placebo according to previous randomization. Wound dressing was applied covering the whole wound area. On the following 6 days, APOSEC and placebo were re-applied daily. At day 7, treatment was terminated, wound closure and scar formation were evaluated, and wounds were closed with a suture. During the whole treatment period, wounds were assessed for the appearance of adverse events, and photographs for planimetric assessment were taken (Fig. 3). For standardization of the planimetric measurements, a pacer (CASTEL-COP-DIGI, CASTEL-L, Novoflex, Germany), ensuring the exact same distance for every picture was used (Supplementary Figure S1).

#### Follow-up phase

Each study participant was asked to return to the clinic to allow evaluation of whether or not adverse events emerged during the whole study period. At 17 days after treatment initiation, study participants returned for a follow-up visit. Sutures were removed, a physical examination was performed, vital signs and adverse events were assessed, and blood samples were taken. (Supplementary Table S3 in the Supplement).

### Authorization and ethics statement

The study was approved by the ethics committee of the Medical University of Vienna, Austria (EK Nr. 1285/2013) and conducted according to the Declaration of Helsinki. This trial was registered in the EU clinical trial register (EudraCT-Number: 2013-000756-17; NCT02284360; AGES INS-480102-0013-007).

### Production of APOSEC and placebo

Blood obtained from each study volunteer at the Austrian Red Cross Blood Transfusion Service of Upper Austria was used to produce autologous APOSEC according to current GMP guidelines. PBMCs were separated from whole blood samples of the participants by density centrifugation using LSM 1077 (Lymphocyte Separation Medium, Lonza, Switzerland). Removal of LSM was achieved by two washing steps using Dulbecco’s phosphate-buffered saline (Lonza, Switzerland). PBMCs were resuspended in phenol red–free CellGro GMP DC medium (CellGenix, Freiburg, Germany) containing no xenogeneic proteins. A sample was drawn for complete blood count to adjust white blood cells to a concentration of 25 × 10^6^ cells/ml. Irradiation with 60 Gy induced apoptosis of PBMCs. By cultivation of apoptotic PBMCs in CellGro GMP DC medium, release of the secretome was achieved. After incubation for 24 h ± 2 h, cells were removed by centrifugation. The supernatant containing the secretome was sterile filtered at a pore size of 0.22 μm. The adequate production of APOSEC was defined by appropriate secretion of the following important cytokines: interleukin (IL)-8 (0–5214 pg/ml), epidermal growth factor (EGF; 25–226 pg/ml), and transforming growth factor-β (TGF-β; 2575–21732 pg/ml).

Lyophilized culture medium not containing any cells (CellGro, CellGenix, Freiburg, Germany) served as placebo.

#### Quality and stability

The raw material, i.e., separated PBMCs, was irradiated with 60 Gy and cultured for 24 h. The supernatant of the cells was obtained and subjected to quality assurance protocols. Quality control of the product was realized in several steps. First, sterility testing of the final product was performed. Second, induction of apoptosis was determined before irradiation and after cultivation of the cells by fluorescence-activated cell sorting analysis using the FITC Annexin V Apoptosis Detection Kit (BD Biosciences, Franklin Lakes, NJ, US). Third, concentrations of IL-8/C × CL8 (C × C-motive-chemokine 8), EGF, and TGF-β were determined with enzyme-linked immunosorbent assay (ELISA) to verify successful production of APOSEC according to GMP definitions. The fourth part of quality control was endotoxin, mycoplasma and sterility testing of the final product. Cell culture supernatant samples were additionally screened for herpes contamination via polymerase chain reaction. AGES approved APOSEC as a test product according to current guidelines of the Austrian Drug Registration and Administration Act (AGES INS-480102-0013-007).

### Evaluation of adverse events

Adverse events were documented if reported by study participants or observed by physicians. Skin alterations were graded using a local tolerability assessment scale (0 = no visible reaction; 1 = faint, minimal erythema; 2 = erythema; 3 = erythema with induration or vesicles; and 4 = severe erythema with induration, vesicles, or bullae or pustules and/or erosion/ulceration). A detailed description of all adverse events can be found in the supplementary (Table S2).

### Evaluation of wound healing progression

**Figure 4:**
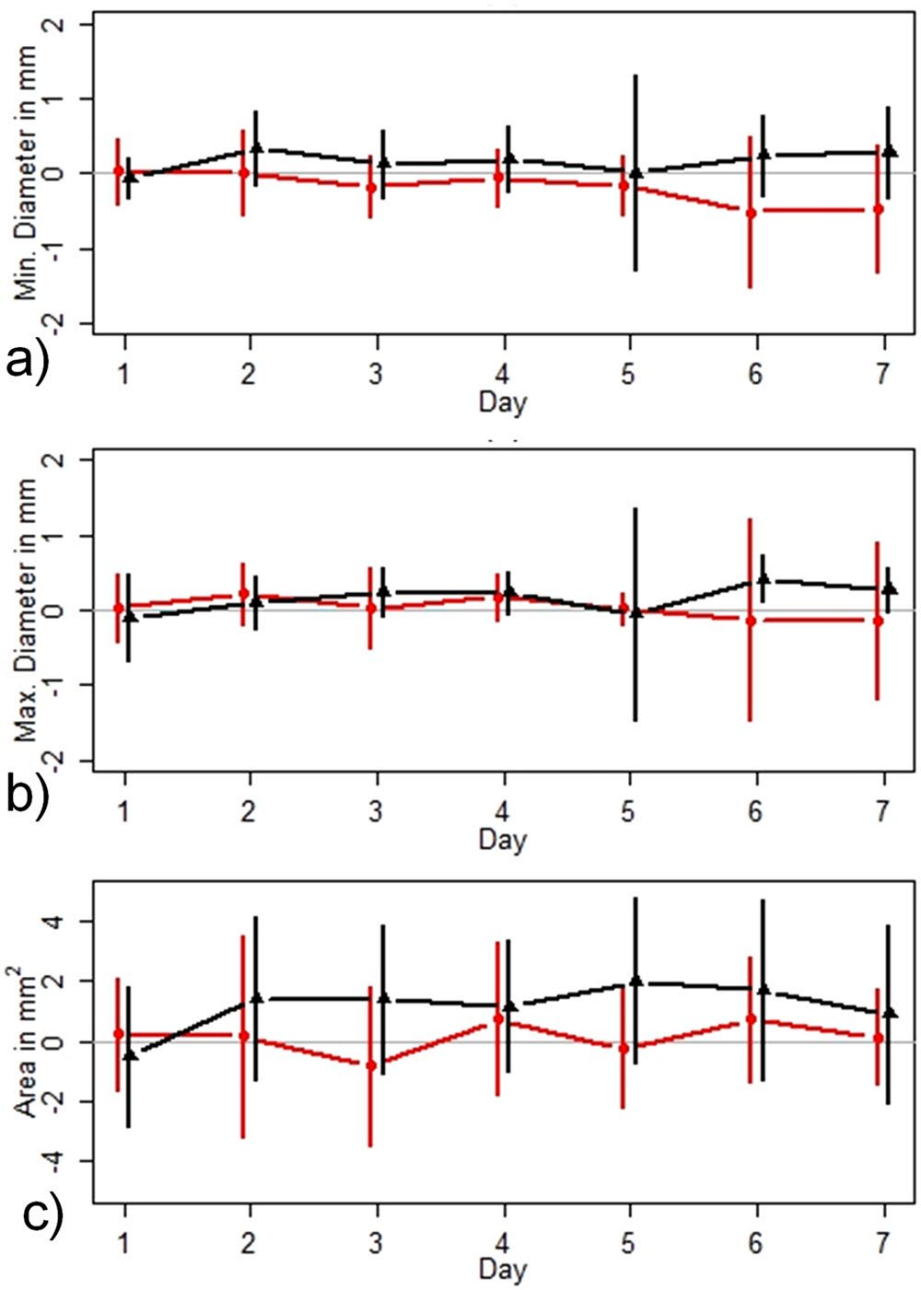
Effect of APOSEC on wound healing. Mean progression as well as the standard deviation (vertical lines) of minimal (**a**) and maximal (**b**) diameters and area (**c**) of the artificial wound during 7 days, represented as the difference of APOSEC – placebo. (red, group A = low-dose group; black, group B = high-dose group).

Progression of wound healing was evaluated by planimetric measurement of photographs, expressed as minimum diameter, maximum diameter, and area measured from day 1 to day 7. (Fig. 4) Measurements were performed using ImageJ version 1.48 v (Wayne Rasband, National Institutes of Health, USA). (Supplementary Tables S4, S5, and S6) Moreover, wound healing and condition were assessed in comparison to prior day (stable, improving, impaired) according to the following criteria: presence or absence of inflammation, presence or absence of exsudate on the dressing, presence or absence of re-epithelialization and presence or absence of undermining and tunneling. Local tolerability was evaluated using the following criteria: (0 = no visible reaction; 1 = faint, minimal erythema; 2 = erythema; 3 = erythema with induration or vesicles; and 4 = severe erythema with induration, vesicles, or bullae or pustules and/or erosion/ulceration).

### Immunohistochemical staining

For microscopic examination, tissue specimens were collected on day 1 and day 7.

Immunohistochemical staining was performed for CD45, keratin 10, factor VIII, and podoplanin. A detailed description of the staining and results can be found in the supplementary material.

### Statistical analysis

Data obtained were evaluated statistically using R version 3.2.1., IBM SPSS Statistics version 23 (SPSS Inc., Chicago, USA), and GraphPad Prism 6 software (GraphPad Software Inc., La Jolla, CA, USA). The analyses were performed for the “as treated” population using descriptive statistics. For the continuous parameters of wound and scar assessment, means, standard deviations as well as medians, quartiles, minima and maxima were calculated separately for the two APOSEC groups and the placebo group as well as for the difference between APOSEC and placebo for all investigated days.

### Data Availability

The datasets generated during and/or analysed during the current study are available from the corresponding author on reasonable request.

## Results

### Study population

In February 2015, fourteen volunteers were assessed for study eligibility, received a case report form (CRF) number, and gave their written informed consent. One was preliminarily excluded before receiving any test treatment, due to a screening failure. Two study participants dropped out due to a deviation in production of APOSEC, and one was excluded because of erythema at the site of placebo application on intact skin at day 0. The proband (CRF 9), excluded before test treatment was included in the 17 screened subjects not meeting inclusion criteria in Fig. 1. The 3 participants excluded due to production deviation and erythema at the placebo treated areal were declined to participate (Fig. 1). The “as treated” population consisted of 10 healthy male study participants.

Supplementary Table S1 describes participant characteristics at the beginning and end of study. At the baseline and end of study visit, study subjects assigned to groups A and B did not show any relevant differences.

### Topical application of APOSEC is safe and well tolerated

The main objective was to monitor for and identify adverse events after topical application of APOSEC. All adverse events were reported by participants or observed by study researchers (from the Department of Clinical Pharmacology of the Medical University of Vienna, Austria) and are shown in (Supplementary Table S2 in the Supplement). All identified events were characterized as mild.

### Wound closure and APOSEC

Due to the short intervention time, we could not demonstrate a further increase in wound closure progression in wounds treated with APOSEC GMP compared to wounds treated with placebo. No wound closure in the artificial wounds was assessed. Figure 3 shows mean time course for maximum wound diameter (A), minimum wound diameter (B), and wound area (C) for the relative difference between verum and placebo measurements separately for group A (red line: 12.5 × 10^6^ PBMC/ml) and B (black line: 25.0 × 10^6^ PBMC/ml). A value below 0 indicates improved wound closure in the APOSEC group as compared to placebo.

## Discussion

In this first clinical prospective phase 1 study utilizing the autologous secretome of PBMCs in humans, we showed that the application of APOSEC is safe and well tolerated in human intact skin, as well as on the open wound area. The secondary endpoint of wound closure was not achieved, which is attributable to the short duration of the study.

This study was performed as a “prerequisite” for the further development of the allogeneic APOSEC product, derived from healthy blood donors in order to treat patients with non-healing wounds. This disease causes in our society an ever increasing financial and psychological burden - for both, patients and the health care system^1^.

In particular, cell-based therapies are a rapidly expanding sector in wound closure treatments. For example, the application of cellular 3D fibroblast constructs (Dermagraft) (Shire Regenerative Medicine, San Diego, CA) received market authorization in multiple countries after Phase 3 trials^24–29^. Another approach has been the use of allogeneic gamma-irradiated cord blood mononuclear cells in a patient with critical limb ischemia, which led to improved wound closure and vascularity^30^.

A similar method was chosen for a clinical trial financed by Macrocure Ltd. In two US Food and Drug Administration (FDA)-approved studies, hypo-osmotic shock-exposed allogeneic PBMCs were injected subcutaneously for the treatment of diabetic and venous foot ulcers^31, 32^ (https://clinicaltrials.gov/ct2/show/NCT01421966).

Both investigations were prematurely terminated because of futility (http://investor.macrocure.com/releasedetail.cfm?ReleaseID=928245).

In contrast to these cell-based therapies, we have concentrated on the biological effects of paracrine factors derived from stressed white blood cells. The supernatant provides a potent cell-free alternative, displaying a possible diminished immunogenicity as compared to cell-based therapy. APOSEC stimulates migration of fibroblasts, keratinocytes, and endothelial cells *in vitro* ^10, 13^, which are crucial elements in the physiology of wound healing. Moreover, APOSEC contains significant amounts of antimicrobial peptides that possess antimicrobial activity against opportunistic skin pathogens, especially *Escherichia coli* and *Pseudomonas aeruginosa* ^23^. With regard to the cataclysmic consequences of bacterial infection for wound regeneration and healing, in severe cases involving non-remediable tissue impairment necessitating amputation, this particular attribute emphasizes the clinical potential of APOSEC ^2^.

Results from a murine wound-healing model, as well as a porcine third-degree burn model have already indicated the effectiveness of topical application of PBMC-derived paracrine factors^10, 14^. Mildner *et al*. showed in this first investigation that the PBMC secretome increases angiogenesis and wound closure in mice^10, 14^. All of these features are most desirable for wound healing, but it is a fact that the PBMC secretome is a mixture of paracrine factors containing multiple pro-angiogenic proteins, lipids, and exosomes^13^. From our point of view, the observed effects are not attributable to a single factor but to the combination of different components of APOSEC. This hypothesis has already been corroborated by Lichtenauer *et al*., who selectively blocked different factors, including matrix metalloproteinase-9 (MMP-9), vascular endothelial growth factor (VEGF), and IL-8, and failed to attenuate the biological activity in selected potency assays^18^. Thus, the identification of a single mechanism of action (MOA) remains challenging because on the one hand, we deal with a complex composition of paracrine factors, and on the other hand, we deal with a plethora of biological effects. Based on our long lasting research in the effect of PBMC secretome (APOSEC) we feel that the search for “the target” or “the MOA” in skin regeneration is not feasible^33^.

Before the “off the shelf” drug substance of allogeneic APOSEC enters regulatory approval, multiple requirements must be met by a drug developer. These are stability studies, development of validated potency assays, and the completion of incremental and repeated dose toxicology studies in two animal species.

All of these manufacturing and regulatory hurdles must be accomplished before a transition into the clinic will become reality. In addition to a positive verdict of the internal reviewer board (IRB), trial registration and approval of national and super national regulatory agencies are mandatory.

Only a proof of concept phase II study will show whether scientific insights generated at our surgical research laboratory will find its translation in the treatment of non-healing wounds.

## Electronic supplementary material

Supplementary
