# Supplementary material for: Safety and tolerability of topically administered autologous, apoptotic PBMC secretome (APOSEC) in dermal wounds: a randomized Phase 1 trial (MARSYAS I﻿)
Source: Sci Rep. 2017 Jul 24;7:6216. doi: 10.1038/s41598-017-06223-x (PMC5524970; doi:10.1038/s41598-017-06223-x)
Supplement: Supplementary file 1 — Supplementary [file 41598_2017_6223_MOESM1_ESM.pdf]

Safety and tolerability of topically administered autologous, apoptotic PBMC secretome (APOSEC™) in dermal wounds: a randomized phase 1 trial (MARSYAS I)

Elisabeth Simader MD[1,2,9,10#], Denise Traxler[1,2#], Mohammad Mahdi Kasiri MD[1,2#], Helmut Hofbauer DPhil[1,2,9,10], Michael Wolzt MD[3], Christoph Glogner[1,2], Angela Storka[12], MD[3,11], Michael Mildner PhD,[4,9], Ghazaleh Gouya MD[3], Alexandra Geusau MD[5], Carola Fuchs RN[3], Claudia Eder RN[3], Alexandra Graf PhD[6], Michaela Schaden MSc[7], Bahar Golabi[4], Marie-Bernadette Aretin DP[11], Susanne Suessner MSc[8], Christian Gabriel MD[13], Walter Klepetko MD[1], Erwin Tschachler MD[4],Hendrik Jan Ankersmit MD*[1,2,9,10]

[#] the authors contributed equally to the study

[1] Division of Thoracic Surgery, Medical University of Vienna, Austria
[2] Christian Doppler Laboratory for Cardiac and Thoracic Diagnosis and Regeneration, Medical University of Vienna, Austria
[3] Department of Clinical Pharmacology, Medical University of Vienna, Austria
[4] Research Division of Biology and Pathobiology of the Skin, Department of Dermatology, Medical University of Vienna, Vienna, Austria.
[5] Division of Immunology, Allergy, and Infectious Diseases, Department of Dermatology, Medical University of Vienna
[6] Section of Medical Statistics, Centre for Medical Statistics, Informatics and Intelligent Systems, Medical University of Vienna, Austria.
[7] Clinical Trials Coordinative Centre, Medical University of Vienna, Austria
[8] Austrian Red Cross Blood Transfusion Service for Upper Austria, Linz, Austria
[9] FFG Project 852748 "APOSEC", Medical University of Vienna, Austria
[10] Aposcience AG, Company No.: 308089, Vienna, Austria
[11] AKH Vienna pharmacy, Medical University of Vienna, Austria
[12] Danubian Hospital, Social and medical center of eastern Vienna, Austria
[13] Ludwig Boltzmann Institute for experimental and clinical traumatology, Austria

***Corresponding Author:**

Hendrik Jan Ankersmit, MD
Division of Thoracic Surgery
Medical University of Vienna
Waehringer Guertel 18-20
1090 Vienna, Austria
Tel: +43-1-4040069770
E-Mail: hendrik.ankersmit@meduniwien.ac.at

Supplementary

Adverse event screening

Participants were examined using laboratory tests, ECG and physical examination.

The **physical examination** included an examination of the abdomen, head, ears, eyes, nose, throat, neck, neurological and psychiatric status, as well as pulmonary, cardiovascular, skeletal, muscular, urogenitary tract; measurement of height and weight, BMI. The vital signs measurement included the assessment of the systolic and diastolic pressure, as well as the pulse rate.
Regarding the general physical examinations, no abnormalities were found at any of the screened subjects.

The **standard 12-lead ECG** (25mm/s and 0.1mV/mm) was recorded after at least 5 minutes rest at screening visit via Siemens Megacart or GE MAC 1200ST. For 5 subjects ECG abnormalities were detected at the screening, but none was regarded as clinical significant by the investigator team.

Hematology parameters included the determination of erythrocytes, leukocytes, hemoglobin, hematocrit, thrombocytes, MCV, MCH, MCHC, PTT and aPTT. The levels were obtained at screening and the follow-up for CRF Nr. 01,02,03,04,05,06,07,08,10,11,12,13,14). No abnormalities were detected at any of the subjects.

Serum chemistry included the determination of sodium, potassium, total protein, albumin, chloride, BUN, creatinine, glucose, ASAT, ALAT, AP and gamma GT. The levels were obtained at screening and the follow-up for CRF Nr. 01, 02, 03, 04, 05, 06, 07, 08, 10, 11, 12, 13, 14). No abnormalities were detected at any of the subjects.

Virology included the determination of HBs Ag, HCV Ab and HIV-1/2 Ab levels. No latent or active infection was detected in any of the screened subjects.

Urine analysis included the determination of pH, leukocytes, nitrite, protein, glucose and blood. No abnormalities were detected at any of the screened subjects regarding the pH

Analysis, moreover all revealed negative results with respect to the parameters measured at the screening.

Supplementary Tables

Supplementary Table S1. Disposition of subjects according to dose group

CRF Nr.	Dose group	Treatment proximal	Treatment distal	Randomization code	Status	Drop-out	Reason
1	A (12.5×10^6 PBMC/ml)	Placebo	Verum	DR2	Drop-out	Day 1	Primary reason for discontinuation is deviation in IMP production (Sponsor decision) Evaluation of test treatment on day 1: Area proximal: 01: faint, minimal erythema. area distal: 03: erythema with induration or vesicles, hives, no itching, no pain, no burning
2	A (12.5×10^6 PBMC/ml)	Verum	Placebo	DR1	Drop-out	Day 1	Primary reason for discontinuation is deviation in IMP production (Sponsor decision) Evaluation of test treatment on day 1: Area proximal: 0: hives without itching, no erythema. Area distal: 0: no visible reaction
3	A (12.5×10^6 PBMC/ml)	Verum*	Placebo*	DR2*	Completed	n.a.	n.a.
4	A (12.5×10^6 PBMC/ml)	Placebo*	Verum*	DR1*	Completed	n.a.	n.a.
5	A (12.5×10^6 PBMC/ml)	Verum	Placebo	DR1	Completed	n.a.	n.a.
6	A (12.5×10^6 PBMC/ml)	Verum	Placebo	DR1	Completed	n.a.	n.a.
7	A (12.5×10^6 PBMC/ml)	Placebo	Verum	DR2	Drop-out	Day 1	Evaluation of test treatment on day 1: Area proximal: 01 faint, minimal erythema
8	A (12.5×10^6 PBMC/ml)	Verum	Placebo	DR1	Completed	n.a.	n.a.
9	B (25.0×10^6 PBMC/ml)	n.a.	n.a.	n.a.	Not included	Preliminary exclusion	Due to a screening failure, the patient was preliminarily excluded before test treatment
10	B (25.0×10^6 PBMC/ml)	Placebo	Verum	DR2	Completed	n.a.	n.a.
11	B (25.0×10^6 PBMC/ml)	Placebo	Verum	DR2	Completed	n.a.	n.a.
12	B (25.0×10^6 PBMC/ml)	Verum	Placebo	DR1	Completed	n.a.	n.a.

Supplementary Table S1. Disposition of subjects according to dose group (continued)

13	B (25.0×10^6 PBMC/ml)	Verum	Placebo	DR1	Completed	n.a.	n.a.
14	B (25.0×10^6 PBMC/ml)	Verum	Placebo	DR1	Completed	n.a.	n.a.

Supplementary Table S1

Summary of all study subjects and their treatment randomization. Dose group A represents the low-dose group. Dose group B was the high-dose group. Verum was applied on the proximal artificial wound and placebo on the distal wound when coded with DR1. DR2 coded participants had an application of verum to the distal wound and placebo to the proximal wound. The double-blinded study randomization was performed by the AKH pharmacy.

*Subjects CRF Nrs. 3 and 4 were treated vice versa from the assigned randomization code; this alteration occurred throughout the study. The outcome of the randomizer web application revealed code DR1 for treatment at distal and code DR2 for treatment at proximal. The AKH pharmacy determined to administer placebo to the randomized location.

Supplementary Table S2. Index of adverse events

			MedDRA Coding											
**CRF#**	**Description**	**Applied IMP**	**LLT**<br>**MedDRA Coding**	**LLT**	**SOC**	**SOC**	**Comment/**<br>**Query**	**Outcome**	**Severity**	**Unexpected**	**Serious**	**Drug treatment**	**Action**	**Relation**
1	Faint, minimal erythema area proximal	Placebo<br>Dose group A	Application site erythema	100<br>030<br>41	Skin and subcutaneous tissue disorders	1004<br>0785	Comment: graded as local tolerability 1	No follow-up	Not assessed due to grading as local tolerability via score			No	Premature study discontinuation[1]	Not assessed due to grading as local tolerability via score
1	Erythema with induration or vesicles, hives no itching, no pain, no burning area distal	Verum<br>Dose group A	Application site erythema	100<br>030<br>41	Skin and subcutaneous tissue disorders	1004<br>0785	Comment: graded as local tolerability 3	No follow-up	Not assessed due to grading as local tolerability via score			No	Premature study discontinuation[1]	Not assessed due to grading as local tolerability via score
2	Hives without itching, no erythema area proximal	Verum<br>Dose group A	Hives	100<br>201<br>97	Skin and subcutaneous tissue disorders	1004<br>0785	Comment: graded as local tolerability 0	No follow-up	Not assessed due to grading as local tolerability via score			No	Premature study discontinuation[1]	Not assessed due to grading as local tolerability via score
4	Redness right upper arm at the patch area	Not assignable to verum or placebo<br>Dose group A	Application site redness	100<br>030<br>58	Skin and subcutaneous tissue disorders	1004<br>0785	Comment: upper right arm	Resolved	Mild	Yes	No	No	No action	Unlikely
7	Sore throat	Not assignable to verum or placebo<br>Dose group A	Sore throat	100<br>413<br>67	Respiratory, thoracic, and mediastinal disorders	1003<br>8738	Comment: Not applicable	Resolved	Mild	Yes	No	No	No action	Unrelated

Supplementary Table S2. Index of adverse events (continued)

7	Erythema in area proximal	Placebo<br>Dose group A	Application site erythema	100 030 41	Skin and subcutaneous tissue disorders	1004 0785	Comment: proximal;<br>Graded as local tolerability grade 1	Resolved	Mild	Yes	No	No	Premature study discontinuation, no further action	Probably (documented as both adverse events and local tolerability effect).
**8**	Distal wound opened and bleeding	Placebo<br>Dose group A	Wound bleeding	100 513 86	Injury, poisoning, and procedural complications	1002 2117	Comment: distal	Resolved	Mild	Yes	No	No	01.05.2015: Cleaning and application of dressing<br>04.05.2015: Photo documentation<br>Assessment: slough dry and application of dressing	Unrelated
8	Two hematomas left upper arm around the puncture site of the local anesthetic	Not assignable to verum or placebo<br>Dose group A	Injection site hematoma	100 553 71	General disorders and administration site conditions	1001 8065	Comment: left upper arm	Resolved	Mild	Yes	No	No	No action	Unrelated
11	Sensitivity of skin by Steri Strips in the dressing area proximal	Placebo<br>Dose group B	Adhesive plaster sensitivity	100 012 90	Skin and subcutaneous tissue disorders	1004 0785	Comment: proximal	Resolved	Mild	Yes	No	No	No action	Unrelated
14	Muscle tension left arm	Not assignable to verum or placebo<br>Dose group B	Muscle tension	100 705 41	Musculoskeletal and connective tissue disorders	1002 8395	Comment: left arm	Resolved	Mild	Yes	No	Transfer to neurologis t	No action	Unrelated
14	Itching distal wound	Placebo<br>Dose group B	Wound itching	100 628 73	Injury, poisoning, and procedural complications	1002 2117	Comment: distal	Resolved	Mild	Yes	No	No	No action	Probably
14	Itching proximal wound	VerumDos e group B	Wound itching	100 628 73	Injury, poisoning, and procedural complications	1002 2117	Comment: proximal	Resolved	Mild	Yes	No	No	No action	Probably

Supplementary Table S2

Summary of all adverse events and attribution to verum and placebo. CRF#: ID of the study participants. In the dose group A, the low dose was applied, and in dose group B, the high dose of APOSEC^{TM} was applied. Tolerability was quantified using a 4-point local tolerability assessment scale (0=no visible reaction; 1=faint, minimal erythema; 2=erythema; 3=erythema with induration or vesicles; 4=severe erythema with induration, vesicles, or bullae or pustules and/or erosion/ulceration).

Supplementary Table S3. Demographic characteristics of the study participants

eCRF Nr.	Visit date	ICF signed	ICF date	Screening Nr.	Birth year	Gender	Ethnicity	Alcohol consumption	Alcohol units [units/week]*	Smoking	Smoking quantity [cigarettes/day]
1	13 Feb 2015	Yes	13 Feb 2015	17	1986	Male	Caucasian	Yes	1	Non-smoker	0
2	09 Feb 2015	Yes	09 Feb 2015	16	1984	Male	Caucasian	No	0	Non-smoker	0
3	26 Feb 2015	Yes	26 Feb 2015	21	1988	Male	Caucasian	Yes	1	Non-smoker	0
4	20 Feb 2015	Yes	20 Feb 2015	18	1989	Male	Caucasian	No	0	Non-smoker	0
5	23 Feb 2015	Yes	23 Feb 2015	19	1994	Male	Caucasian	No	0	Non-smoker	0
6	24 Feb 2015	Yes	24 Feb 2015	20	1989	Male	Caucasian	Yes	n.a.	Non-smoker	0
7	02 Mar 2015	Yes	02 Mar 2015	22	1987	Male	Caucasian	Yes	2	Smoker	20
8	06 Mar 2015	Yes	06 Mar 2015	25	1990	Male	Caucasian	Yes	3	Non-smoker	0
10	02 Mar 2015	Yes	02 Mar 2015	23	1985	Male	Caucasian	No	0	Ex-smoker	20
11	25 Mar 2015	Yes	25 Mar 2015	27	1989	Male	Caucasian	Yes	1	Smoker	4
12	27 Mar 2015	Yes	27 Mar 2015	28	1983	Male	Caucasian	No	0	Non-smoker	0
13	09 Apr 2015	Yes	09 Apr 2015	30	1987	Male	Caucasian	No	0	Smoker	7
14	09 Apr 2015	Yes	09 Apr 2015	29	1988	Male	Caucasian	No	0	Smoker	10

Supplementary Table S3

Summary of all volunteers, depicting anonymization ID during the study (eCRF Nr.), date of signing the informed written consent (ICF), screening number, age, gender, ethnicity, alcohol consumption, and smoking characteristics.

*1 unit equals half a liter of beer, 200 mL wine, or 50 mL of spirits.

Supplementary Table S4. Time course of the maximum wound diameter for both dose groups

Day	Group	Statistic	Verum	Placebo	Difference
Day 1	Group A	Mean (SD)	5.14 (0.45)	5.11 (0.28)	0.03 (0.46)
		Median (Q1 to Q3)	4.94 (4.93 to 4.98)	5.07 (4.92 to 5.24)	0.02 (-0.09 to 0.09)
		Min to Max	4.91 to 5.94	4.82 to 5.52	-0.59 to 0.7
	Group B	Mean (SD)	5.21 (0.31)	5.31 (0.37)	-0.09 (0.58)
		Median (Q1 to Q3)	5.09 (5.07 to 5.19)	5.08 (5.08 to 5.49)	-0.11 (-0.4 to 0.11)
		Min to Max	4.97 to 5.75	5.01 to 5.88	-0.81 to 0.74
Day 2	Group A	Mean (SD)	5.24 (0.5)	5.03 (0.19)	0.21 (0.42)
		Median (Q1 to Q3)	5.13 (4.95 to 5.66)	5.03 (5.01 to 5.14)	0.4 (-0.08 to 0.52)
		Min to Max	4.64 to 5.84	4.73 to 5.24	-0.37 to 0.6
	Group B	Mean (SD)	5.08 (0.07)	4.97 (0.37)	0.11 (0.36)
		Median (Q1 to Q3)	5.07 (5.05 to 5.07)	5 (4.86 to 5.24)	0.14 (-0.17 to 0.2)
		Min to Max	5 to 5.2	4.41 to 5.35	-0.28 to 0.64
Day 3	Group A	Mean (SD)	4.9 (0.51)	4.88 (0.15)	0.03 (0.53)
		Median (Q1 to Q3)	5.09 (4.45 to 5.16)	4.85 (4.79 to 4.99)	0.03 (-0.25 to 0.37)
		Min to Max	4.3 to 5.52	4.7 to 5.06	-0.69 to 0.67
	Group B	Mean (SD)	5.06 (0.11)	4.81 (0.31)	0.25 (0.32)
		Median (Q1 to Q3)	5.04 (5.01 to 5.05)	4.83 (4.66 to 4.93)	0.32 (0.14 to 0.39)
		Min to Max	4.97 to 5.25	4.4 to 5.24	-0.23 to 0.64
Day 4	Group A	Mean (SD)	4.94 (0.35)	4.76 (0.18)	0.17 (0.3)
		Median (Q1 to Q3)	4.94 (4.8 to 5.22)	4.78 (4.62 to 4.89)	0.25 (-0.09 to 0.39)
		Min to Max	4.43 to 5.29	4.55 to 4.97	-0.19 to 0.51
	Group B	Mean (SD)	4.86 (0.06)	4.64 (0.27)	0.23 (0.29)
		Median (Q1 to Q3)	4.85 (4.84 to 4.86)	4.58 (4.43 to 4.79)	0.38 (0.02 to 0.43)
		Min to Max	4.81 to 4.96	4.37 to 5.02	-0.18 to 0.48
Day 5	Group A	Mean (SD)	4.66 (0.33)	4.65 (0.21)	0.02 (0.22)
		Median (Q1 to Q3)	4.59 (4.44 to 4.87)	4.6 (4.47 to 4.77)	-0.07 (-0.16 to 0.12)
		Min to Max	4.29 to 5.12	4.45 to 4.94	-0.16 to 0.35
	Group B	Mean (SD)	4.39 (1.06)	4.44 (0.44)	-0.05 (1.42)
		Median (Q1 to Q3)	4.87 (4.76 to 4.89)	4.56 (4.08 to 4.57)	0.37 (0.32 to 0.68)
		Min to Max	2.49 to 4.93	3.95 to 5.05	-2.56 to 0.92
Day 6	Group A	Mean (SD)	4.48 (1.3)	4.61 (0.15)	-0.14 (1.35)
		Median (Q1 to Q3)	4.86 (4.66 to 5.32)	4.57 (4.54 to 4.73)	0.44 (0.12 to 0.52)
		Min to Max	2.21 to 5.33	4.42 to 4.8	-2.52 to 0.76
	Group B	Mean (SD)	4.82 (0.13)	4.41 (0.31)	0.42 (0.31)
		Median (Q1 to Q3)	4.77 (4.72 to 4.9)	4.33 (4.16 to 4.63)	0.56 (0.08 to 0.68)
		Min to Max	4.71 to 5.01	4.09 to 4.82	0.08 to 0.68
Day 7	Group A	Mean (SD)	4.31 (1.19)	4.45 (0.3)	-0.15 (1.04)
		Median (Q1 to Q3)	4.8 (4.36 to 4.98)	4.45 (4.21 to 4.49)	0.22 (-0.13 to 0.53)
		Min to Max	2.24 to 5.15	4.18 to 4.93	-1.94 to 0.59
	Group B	Mean (SD)	4.61 (0.12)	4.33 (0.24)	0.28 (0.29)
		Median (Q1 to Q3)	4.66 (4.48 to 4.71)	4.41 (4.13 to 4.46)	0.3 (0.2 to 0.35)
		Min to Max	4.48 to 4.72	4.05 to 4.62	-0.14 to 0.67

Supplementary Table S4

Descriptive statistics of the maximum diameter. Mean diameter and standard deviation (SD) as well as median, quantiles, minima and maxima in mm for application days 1–7, separated for dose group A (low-dose group) and group B (high-dose group).

Supplementary Table S5. Time course of the minimum wound diameter for both dose groups

Day	Group	Statistic	Verum	Placebo	Difference
Day 1	Group A	Mean (SD)	4.31 (0.3)	4.28 (0.41)	0.03 (0.43)
		Median (Q1 to Q3)	4.26 (4.25 to 4.5)	4.47 (4.13 to 4.51)	-0.01 (-0.01 to 0.12)
		Min to Max	3.88 to 4.66	3.63 to 4.67	-0.59 to 0.63
	Group B	Mean (SD)	4.6 (0.11)	4.67 (0.24)	-0.07 (0.27)
		Median (Q1 to Q3)	4.56 (4.55 to 4.69)	4.69 (4.54 to 4.78)	0 (-0.04 to 0.01)
		Min to Max	4.47 to 4.74	4.36 to 4.99	-0.52 to 0.2
Day 2	Group A	Mean (SD)	4.29 (0.62)	4.28 (0.09)	0.01 (0.57)
		Median (Q1 to Q3)	4.07 (3.82 to 4.66)	4.33 (4.27 to 4.33)	-0.2 (-0.41 to 0.31)
		Min to Max	3.72 to 5.19	4.13 to 4.35	-0.51 to 0.86
	Group B	Mean (SD)	4.38 (0.16)	4.04 (0.4)	0.34 (0.49)
		Median (Q1 to Q3)	4.48 (4.25 to 4.49)	4.01 (3.77 to 4.25)	0.48 (0.27 to 0.48)
		Min to Max	4.18 to 4.52	3.58 to 4.61	-0.43 to 0.9
Day 3	Group A	Mean (SD)	4.04 (0.56)	4.21 (0.22)	-0.18 (0.4)
		Median (Q1 to Q3)	3.86 (3.54 to 4.62)	4.32 (4.13 to 4.37)	-0.32 (-0.46 to 0.23)
		Min to Max	3.53 to 4.64	3.86 to 4.39	-0.6 to 0.27
	Group B	Mean (SD)	4.21 (0.22)	4.08 (0.39)	0.13 (0.45)
		Median (Q1 to Q3)	4.24 (4.2 to 4.31)	3.82 (3.79 to 4.33)	0.02 (-0.09 to 0.52)
		Min to Max	3.84 to 4.44	3.79 to 4.65	-0.45 to 0.65
Day 4	Group A	Mean (SD)	4.03 (0.59)	4.09 (0.29)	-0.06 (0.38)
		Median (Q1 to Q3)	3.88 (3.82 to 4.59)	4.15 (3.83 to 4.19)	0.05 (-0.33 to 0.14)
		Min to Max	3.22 to 4.63	3.78 to 4.49	-0.56 to 0.4
	Group B	Mean (SD)	4.17 (0.3)	3.98 (0.34)	0.19 (0.45)
		Median (Q1 to Q3)	4.2 (4.11 to 4.34)	3.8 (3.8 to 4)	0.4 (-0.1 to 0.48)
		Min to Max	3.7 to 4.48	3.72 to 4.56	-0.45 to 0.62
Day 5	Group A	Mean (SD)	3.85 (0.42)	4.01 (0.25)	-0.16 (0.39)
		Median (Q1 to Q3)	3.6 (3.57 to 4.27)	3.86 (3.86 to 4.1)	-0.27 (-0.4 to -0.14)
		Min to Max	3.46 to 4.35	3.84 to 4.41	-0.5 to 0.49
	Group B	Mean (SD)	3.69 (0.93)	3.69 (0.38)	0 (1.31)
		Median (Q1 to Q3)	3.99 (3.66 to 4.34)	3.6 (3.47 to 3.66)	0.33 (0.06 to 0.89)
		Min to Max	2.1 to 4.36	3.37 to 4.33	-2.23 to 0.97
Day 6	Group A	Mean (SD)	3.44 (1.02)	3.95 (0.16)	-0.52 (1.01)
		Median (Q1 to Q3)	3.4 (3.35 to 4.14)	4.03 (3.91 to 4.04)	-0.35 (-0.51 to 0.05)
		Min to Max	1.82 to 4.47	3.7 to 4.09	-2.21 to 0.43
	Group B	Mean (SD)	3.93 (0.24)	3.69 (0.42)	0.24 (0.53)
		Median (Q1 to Q3)	3.99 (3.81 to 4.07)	3.66 (3.34 to 3.71)	0.15 (-0.13 to 0.73)
		Min to Max	3.58 to 4.18	3.34 to 4.38	-0.39 to 0.84
Day 7	Group A	Mean (SD)	3.41 (1.06)	3.89 (0.42)	-0.47 (0.84)
		Median (Q1 to Q3)	3.62 (3.3 to 3.96)	3.95 (3.65 to 3.96)	-0.07 (-0.33 to 0)
		Min to Max	1.69 to 4.5	3.37 to 4.5	-1.96 to 0
	Group B	Mean (SD)	3.91 (0.34)	3.62 (0.35)	0.29 (0.61)
		Median (Q1 to Q3)	3.94 (3.65 to 4.2)	3.52 (3.48 to 3.61)	0.33 (-0.03 to 0.72)
		Min to Max	3.49 to 4.27	3.29 to 4.22	-0.57 to 0.98

Supplementary Table S5

Descriptive statistics of the minimum diameter. Mean diameter and standard deviation (SD) as well as median, quantiles, minima and maxima in mm for application days 1–7, separated for dose group A (low-dose group) and group B (high-dose group).

.

Supplementary Table S6. Time course of the wound area for both dose groups

Day	Group	Statistic	Verum	Placebo	Difference
Day 1	Group A	Mean (SD)	16.88 (2.15)	16.64 (1.09)	0.25 (1.87)
		Median (Q1 to Q3)	16.32 (16.19 to 17.69)	16.8 (16.16 to 17.6)	0.09 (0.03 to 1.31)
		Min to Max	14.2 to 20.02	15.01 to 17.62	-2.6 to 2.4
	Group B	Mean (SD)	18.5 (1.11)	19.01 (1.78)	-0.51 (2.36)
		Median (Q1 to Q3)	18.55 (17.61 to 18.56)	18 (17.89 to 20.93)	0.32 (-2.38 to 0.56)
		Min to Max	17.52 to 20.28	17.29 to 20.96	-3.44 to 2.39
Day 2	Group A	Mean (SD)	16.51 (3.8)	16.36 (1)	0.15 (3.34)
		Median (Q1 to Q3)	14.32 (13.84 to 18.93)	16.44 (16.27 to 16.72)	-0.49 (-2.6 to 2.66)
		Min to Max	13.45 to 22.03	14.81 to 17.56	-3.27 to 4.47
	Group B	Mean (SD)	16.48 (0.94)	15.05 (2.48)	1.43 (2.71)
		Median (Q1 to Q3)	15.89 (15.79 to 17.48)	14.04 (13.62 to 16.07)	1.85 (1.41 to 3.07)
		Min to Max	15.71 to 17.53	12.64 to 18.88	-3.09 to 3.91
Day 3	Group A	Mean (SD)	14.71 (3.13)	15.54 (0.93)	-0.83 (2.65)
		Median (Q1 to Q3)	13.71 (12.52 to 17.97)	15.53 (14.85 to 16.04)	-1.14 (-3.17 to 1.16)
		Min to Max	11.32 to 18.05	14.49 to 16.81	-3.52 to 2.52
	Group B	Mean (SD)	15.74 (1.32)	14.33 (2.15)	1.41 (2.47)
		Median (Q1 to Q3)	16.19 (15.82 to 16.59)	13.58 (13.53 to 14.56)	2.03 (-0.13 to 3.1)
		Min to Max	13.45 to 16.63	12.13 to 17.84	-2.02 to 4.06
Day 4	Group A	Mean (SD)	15.14 (3.11)	14.41 (1.67)	0.73 (2.54)
		Median (Q1 to Q3)	13.09 (13.06 to 18.22)	13.88 (13.82 to 14.3)	-0.31 (-0.79 to 0.98)
		Min to Max	12.5 to 18.85	12.81 to 17.24	-1.24 to 5.03
	Group B	Mean (SD)	15.1 (0.98)	13.95 (2)	1.15 (2.2)
		Median (Q1 to Q3)	15.4 (15.02 to 15.47)	13.13 (12.78 to 13.61)	1.79 (0.75 to 2.69)
		Min to Max	13.5 to 16.11	12.75 to 17.48	-2.46 to 2.98
Day 5	Group A	Mean (SD)	13.51 (2.55)	13.76 (1.45)	-0.25 (2)
		Median (Q1 to Q3)	12.19 (11.95 to 15.54)	13.23 (13.04 to 13.76)	-0.69 (-1.28 to -0.35)
		Min to Max	10.97 to 16.88	12.54 to 16.23	-2.07 to 3.12
	Group B	Mean (SD)	14.69 (1.5)	12.68 (2.61)	2.01 (2.77)
		Median (Q1 to Q3)	15.04 (14.26 to 15.92)	11.99 (11.51 to 12.63)	2.75 (-0.32 to 3.05)
		Min to Max	12.31 to 15.92	10.2 to 17.06	-1.14 to 5.72
Day 6	Group A	Mean (SD)	13.95 (2.89)	13.21 (1.19)	0.74 (2.09)
		Median (Q1 to Q3)	12.22 (11.85 to 16.67)	13.1 (12.87 to 13.35)	0.13 (-0.65 to 2.48)
		Min to Max	11.5 to 17.5	11.72 to 15.02	-1.6 to 3.32
	Group B	Mean (SD)	14.05 (1.5)	12.33 (2.33)	1.72 (3.02)
		Median (Q1 to Q3)	14.12 (13.29 to 14.18)	11.9 (11.2 to 12)	2.09 (0.29 to 2.22)
		Min to Max	12.29 to 16.36	10.25 to 16.3	-2.12 to 6.11
Day 7	Group A	Mean (SD)	13.12 (2.8)	12.98 (2.5)	0.14 (1.58)
		Median (Q1 to Q3)	12.11 (11.97 to 14.28)	12.96 (11.82 to 13.03)	0.37 (-0.99 to 1.25)
		Min to Max	9.94 to 17.3	10.15 to 16.93	-1.88 to 1.96
	Group B	Mean (SD)	12.94 (1.62)	12.02 (1.66)	0.92 (2.98)
		Median (Q1 to Q3)	12.58 (12.1 to 13.97)	11.55 (11.08 to 12.51)	1.5 (-1.57 to 2.42)
		Min to Max	10.94 to 15.1	10.32 to 14.63	-2.53 to 4.78

Supplementary Table S6

Descriptive statistics of the wound area in mm^2. Mean area and standard deviation (SD) as well as median, quantiles, minima and maxima in mm^2 for application days 1–7, separated for dose group A (low-dose group) and group B (high-dose group).

Supplementary Methods

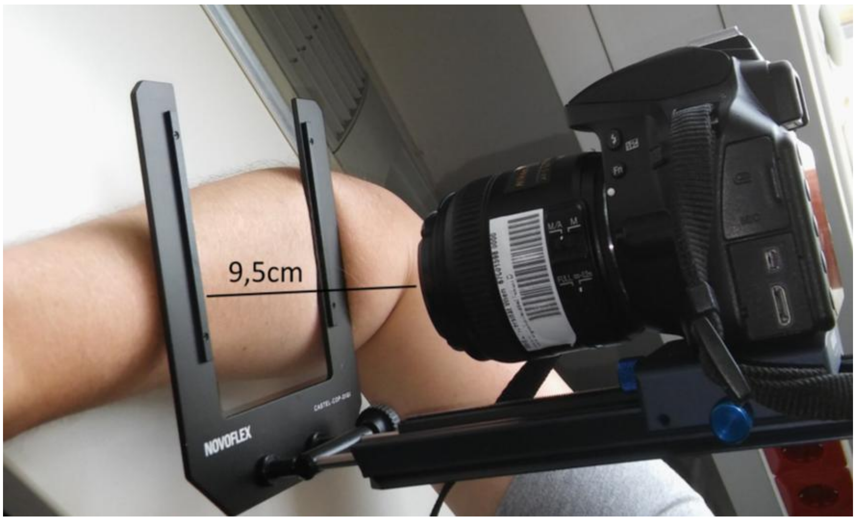

Supplementary Fig. S1

Depiction of the pacer (CASTEL-COP-DIGI, CASTEL-L, Novoflex, Germany) with the camera, in a re-enactment. The distance between the camera lens and the skin amounts to a constant 9,5cm.

Immunohistochemical staining

The specimen taken at day 1 before initiation of therapy depicts a baseline value. A punch biopsy was performed on day 1 (4mm) and day 7 (6mm).The tissue specimens were gathered, by the same surgeon. The depth of the biopsy was defined by the depth of the metal blade (7mm), as the granulation tissue has formed a depth of 7mm was enough to acquire the wound in its entirety on day 7. The tissue specimens were cut in half and one part was prepared for cryosection and the second part for paraffin-embedding. The samples were put into a sterile plastic tube and immediately frozen (for cryosection) or kept in formaldehyde 7.5% for 24 hours. After 24h, sections were prepared for paraffin embedding, by standardized protocols of the Department of Dermatology (Medical University of Vienna). Immunohistochemical staining was performed according to the manufacturer's protocol using the Avidin Biotin Peroxidase complex technique. CD45 (ab10558, Abcam, Cambridge, UK; dilution: 1:100) was stained on frozen sections and keratin 10 (PRB-159P, Covance Research Products Inc., Denver, PA, USA; dilution: 1:1000), factor VIII (A0082, DAKO, Santa Clara, CA, USA; dilution: 1:1000), and podoplanin (clone: D2-40; 322M-15; Cell Marque Corporation, Rocklin, CA, USA; dilution: 1:50) were stained on formalin-fixed, paraffin-embedded sections. In brief, frozen tissue was embedded in OCT prior to sectioning and

stored at -80°C. Tissue specimens were cut into sections 6–8 µm thick and fixed using 4% paraformaldehyde. Formalin-fixed, paraffin-embedded tissue specimens were cut into sections 4–6 µm thick and deparaffinized. The following steps were conducted on frozen as well as formalin-fixed, paraffin-embedded sections. Briefly, heat-mediated antigen retrieval was performed using citrate buffer, pH 6.0. Endogenous peroxidase activity was quenched with 0.3% hydrogen peroxide. Sections were incubated with the appropriate primary antibody overnight at 4°C, followed by incubation with either anti-IgG mouse or anti-IgG rabbit secondary antibody (RPN1001V, Chalfont St. Giles, GB; BA-1000, Vector Laboratories, Burlingame, CA, USA) diluted in 10% sheep or goat normal serum (sc-2488, Santa Cruz Biotechnology Inc., Dallas, TX, USA; X0907, DAKO, Santa Clara, CA, USA) for 30 min at room temperature. Slides were then incubated with ABC reagent (PK 4000, Vector Laboratories, Burlingame, CA, USA) for 30 min at room temperature. The reaction was visualized with AEC substrate (K3469, DAKO, Santa Clara, CA, USA) under the microscope and counterstained with hematoxylin (1.09253.500, Merck, Darmstadt, Germany). As negative controls, the primary antibody was omitted. Additionally, on formalin-fixed, paraffin-embedded sections, hematoxylin–eosin staining was performed according to a standard protocol.

Digital scanning of tissue sections was performed using an automated scanning microscope, TissueFAXs (TissueGnostics, Vienna, Austria). Tissue sections were rated by a blinded observer. Tissue sections from day 7 were divided into transition, wound, and healthy zones. The number of factor VIII–positive or podoplanin-positive vessels per cm^2 (MVD) was counted at a magnification level of 20x. The effect of MVD by APOSEC low dose, APOSEC high dose, or placebo was evaluated at day 7 in wound area (as fold increase to day 1). The number of $CD45^+$ cells per high power field was counted at a magnification level of 20× in wound area (as fold increase to day 1) and the percentage of $CD45^+$ cells was calculated.

Factor VIII APOSEC low dose vs. placebo (median fold increase [range]) (0.67 [0.20; 0.93] vs. 1.24 [0.09; 3.33], p=0.44), Factor VIII APOSEC high dose vs. placebo (median [range]) (1.01 [0.33; 1.48] vs. 0.84 [0.17; 1.49, p=0.63). No podoplanin vessels were found in the tissue specimens. No K10 positive epidermal layer in the wound area was found in any patient at day 7.CD45 APOSEC low dose vs. placebo (median fold increase [range]) (4.03 [1.15; 8.92] vs. 2.09 [0.77; 3.87], p=0.38), CD45 APOSEC high dose vs. placebo (median [range]) (1.58 [0.44; 2.72] vs. 1.48 [0.21; 2.00, p=0.48)
